# Comparative Evaluation of DeepLabCut Convolutional Neural Network Architectures for High-Precision Markerless Tracking in the Mouse Staircase Test

**DOI:** 10.3390/bioengineering13020215

**Published:** 2026-02-13

**Authors:** Valentin Fernandez, Landoline Bonnin, Afsaneh Gaillard, Christine Fernandez-Maloigne

**Affiliations:** 1 LNEC INSERM U1084, University of Poitiers, 86073 Poitiers, France; valentin.fernandez@univ-poitiers.fr (V.F.); afsaneh.gaillard@univ-poitiers.fr (A.G.); 2 LMA CNRS 7348, University of Poitiers, 86073 Poitiers, France; landoline.bonin@univ-poitiers.fr; 3 XLIM CNRS 7252, University of Poitiers, 86073 Poitiers, France

**Keywords:** DeepLabCut, convolutional neural networks (CNN), markerless tracking, staircase test, pose estimation, kinematic analysis, motor cortex lesion, neurobehavioural assessment

## Abstract

Precise quantification of fine motor behaviour is essential for understanding neural circuit function and for evaluating therapeutic interventions in neurological disorders. While markerless pose estimation frameworks such as DeepLabCut (DLC) have transformed behavioural phenotyping, the choice of convolutional neural network (CNN) backbone has a critical impact on tracking performance, particularly in tasks involving small distal joints and frequent occlusions. In this study, we present the first systematic comparison of nine CNN architectures implemented in DLC for lateral-view analysis of skilled reaching movements in the Montoya Staircase test, a gold-standard assay for forelimb dexterity in rodent models of stroke and neurodegenerative disease. Using a dataset comprising both control and primary motor cortex (M1)–lesioned mice, we evaluated model performance across six key dimensions: spatial accuracy (RMSE, PCK@5 px), mean average precision (mAP), robustness to occlusions, inference speed, and GPU memory usage. Our results demonstrate that multi-scale DLCRNet architectures substantially outperform conventional backbones. DLCRNet_ms5 achieved the highest overall accuracy, while DLCRNet_stride16_ms5 provided the most favourable balance between precision and computational efficiency. These findings provide practical methodological guidance for neuroscience laboratories and highlight the importance of CNN architecture selection for the reliable quantification of fine motor behaviour in preclinical research.

## 1. Introduction

Quantifying fine-scale motor behaviour represents a major challenge in contemporary neuroscience, as it directly links neural circuits function to observable behavioural outcomes. Accurate behavioural measurements are essential for characterizing disease progression and for evaluating therapeutic interventions in both preclinical and clinical contexts [1]. This need is particularly acute in neurological disorders, including neurodegenerative diseases such as Alzheimer’s and Parkinson’s disease, as well as traumatic brain injury and stroke, where motor impairments constitute key indicators of underlying neural dysfunction [2,3,4].

Traditional approaches to behavioural assessment rely heavily on manual or semi-quantitative scoring of video recordings. Although widely used, these methods suffer from intrinsic limitations, including observer bias, low temporal resolution and poor inter-rater reliability. Such constraints become especially problematic when attempting to detect subtle motor deficits or fine kinematic changes, which are often critical for early diagnosis or for assessing recovery. Consequently, there is a growing demand for automated, objective and high-resolution behavioural analysis tools capable of capturing movements at the millimetric scale [1,5,6].

Markerless pose estimation frameworks have emerged as powerful solutions to these challenges. Among them, DeepLabCut (DLC) has become a widely adopted tool for high-precision tracking of anatomical landmarks across species and experimental paradigms [6,7,8]. Since its introduction, in 2018 by Mathis et al. in [6], DLC has enabled robust kinematic analyses in complex behavioural tasks, including skilled reaching assays and the Montoya staircase test [4,9,10]. These paradigms require precise tracking of distal joints and fine digit movements, making them particularly sensitive benchmarks for pose estimation performance [2,11].

Despite the widespread adoption of DLC, tracking accuracy and robustness strongly depend on the convolutional neural network (CNN) architecture used as the backbone for pose estimation [12,13]. Different architectures introduce distinct trade-offs between spatial precision, robustness to occlusions, inference speed and computational costs [7,14]. Over recent years, multiple CNN backbones have been integrated into the DLC framework, including classical ResNet variants [15], lightweight architectures such as MobileNet and EfficientNet [16], and more specialized designs such as DLCRNet [17]. While each of these architectures has demonstrated advantages in specific contexts, their relative performance remains highly task-dependent.

The Montoya Staircase test represents a particularly demanding scenario for pose estimation. Accurate analysis of this task requires tracking fine digit movements and pellet interactions under frequent occlusions, small inter-keypoint distances, and highly variable limb trajectories [3,9,10]. In such conditions, suboptimal architectural choices can lead to reduced spatial resolution, loss of critical keypoints, or unstable trajectories, ultimately compromising the reliability of downstream kinematic analyses [4].

Although CNN architecture selection is a critical determinant of pose estimation performance, systematic comparisons within the DeepLabCut framework remain scarce. Most existing studies rely on a limited set of architectures or evaluate performance primarily in relatively simple locomotion or social interaction paradigms [17], leaving an important methodological gap for researchers studying complex, fine motor behaviours.

In this study, this gap is addressed by presenting a comprehensive comparison of nine CNN architectures implemented in DeepLabCut for lateral-view tracking of forelimb movements during the Montoya Staircase test. Using a standardized training and assessment pipeline, we benchmark these architectures across multiple performance dimensions, including spatial accuracy, robustness to occlusions and computational efficiency.

By evaluating performance in both control mice and animals with primary motor cortex (M1) lesions, we provide practical guidance for selecting appropriate CNN backbones for high-precision behavioural quantification. Our results aim to support the standardization of pose estimation methodologies and to facilitate rigorous kinematic analysis in preclinical neuroscience research.

## 2. Materials and Methods

### 2.1. Animal Subjects and Behavioural Paradigm

All experiments were conducted using adult C57BL/6 mice aged 8–12 weeks. Animals were housed under standard laboratory conditions, with a 12 h light–dark cycle, and had ad libitum access to food and water. All experimental procedures were approved by the appropriate local ethics committee (APAFIS authorization n° 43824-2023061416402730 v4) and were performed in accordance with European Union guidelines for animal research.

The experimental dataset comprised both control and primary motor cortex (M1)-lesioned mice (12 animals per group), recorded across multiple behavioural sessions (3 sessions per animal). These recordings encompassed a broad range of successful and unsuccessful reaching attempts to ensure representative sampling of forelimb kinematics.

The Montoya staircase test was employed as the behavioural paradigm [2]. This task uses a dual staircase apparatus in which mice must retrieve food pellets from narrow steps using precise forelimb movements. This task generates complex kinematic patterns involving digit coordination, reach planning and grasp execution, making it particularly suitable for evaluating pose estimation algorithms under challenging conditions characterized by frequent occlusions and the need for fine motor control demands.

### 2.2. Video Acquisition and Preprocessing

Behavioural sessions were recorded using a high-speed digital camera positioned laterally to the staircase apparatus, ensuring consistent visualization of reaching movements. Video recordings were captured at 120 frames per second with a spatial resolution of 1280 × 1024 pixels. A diffused LED lighting system was used throughout all recording sessions to maintain stable lighting conditions and minimize shadows.

**Figure 1:**
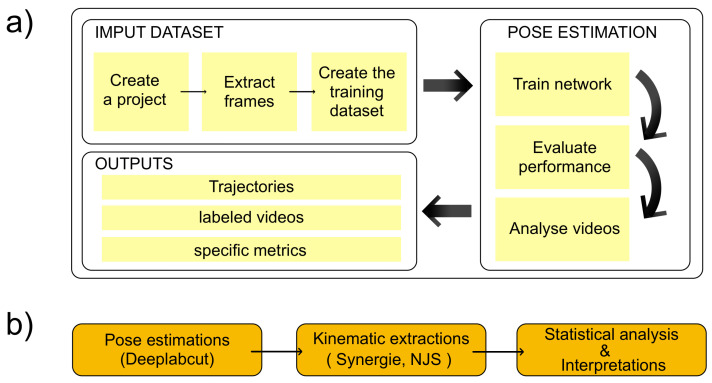
Pipeline for pose estimation using DeepLabCut. (**a**) Input dataset processing steps. (**b**) Model training, inference, and output analysis workflow.

Following acquisition, videos were processed using established protocols to extract individual frames for subsequent analysis. Frames exhibiting motion blur or poor visibility were excluded from further processing to maintain data quality. This preprocessing step ensured that only high-quality images were used for model training and evaluation. The overall analysis pipeline is illustrated in Figure 1.

### 2.3. Keypoint Annotation and Training Procedure

A representative subset of video frames was selected for manual annotation using the DeepLabCut labelling interface. A comprehensive annotation protocol was developed to identify key anatomical landmarks essential for the analysis of forelimb kinematics. In total, 2400 frames representing a wide range of movement patterns were manually annotated by three trained researchers. Annotated frames were selected to ensure coverage of different movement phases, including reach initiation, pellet grasping, and retrieval, as well as both successful and unsuccessful trials. To ensure annotation consistency, inter-rater reliability was assessed on an overlapping subset of annotated frames yielding a mean pixel deviation of 2.073 ± 0.6 pixels across keypoints. In addition, over 93% of keypoint annotations differed by less than 3 pixels between raters, indicating high annotation consistency.

All models were trained using DeepLabCut version 2.X under identical experimental conditions to ensure a fair comparison across architectures. Training and validation sets were created using a fixed split that preserved variability in behavioural states across both partitions.

Data augmentation procedures were applied to improve model generalization and to reduce overfitting to specific postures or lighting conditions. Augmentations included random rotations (±15°), contrast adjustments (±20%), and minor spatial shifts (±5 pixels), which reflect natural variability in animal positioning, camera alignment, and illumination across recording sessions. These transformations preserve the underlying kinematic structure while increasing robustness to experimental variability. After data augmentation, a total of 3000 frames were used for model training, with an 80/20 split between the training and validation sets.

Training was performed using the Adam optimizer, with an initial learning rate adjusted for each architecture based on preliminary stability tests. All models were trained until convergence of the validation loss using identical early-stopping criteria to ensure fair comparison across architectures. Training was terminated when no improvement in validation loss was observed for a predefined number of consecutive epochs. All experiments were conducted on a dedicated GPU workstation (NVIDIA Quadro T1000), ensuring that runtime differences between architectures reflected model characteristics rather than hardware variability.

**Figure A1:**
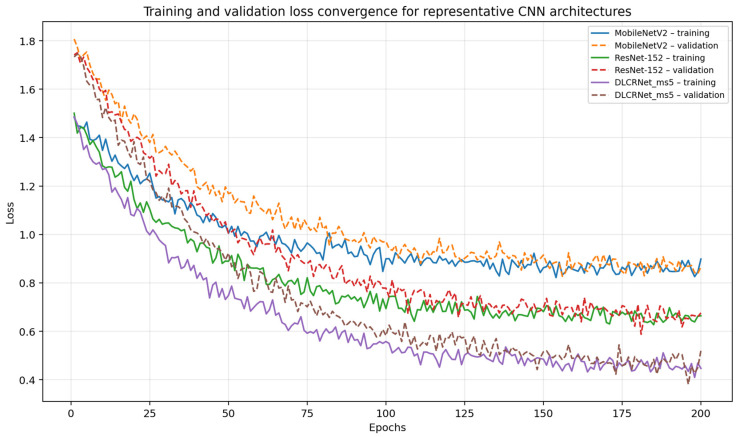
Training and validation loss curves for representative CNN architectures (MobileNetV2, ResNet-152, and DLCRNet_ms5).

Training and validation loss curves for representative architectures (MobileNetV2, ResNet-152, and DLCRNet_ms5) are provided in Appendix A Figure A1, showing stable convergence and the absence of overfitting across models.

### 2.4. CNN Architectures Evaluated

Nine convolutional neural network architectures compatible with DeepLabCut were evaluated: ResNet-50, ResNet-101, ResNet-152, MobileNetV2 [8], EfficientNet-B0, EfficientNet-B3 [9], DLCRNet_ms5, DLCRNet_stride16_ms5, and DLCRNet_stride32_ms5 (Table 1). These architectures were selected to represent the main design strategies currently supported within the DeepLabCut framework: classical deep residual networks, lightweight efficiency-oriented models, and specialized multi-scale architectures.

Other DLC-compatible variants were not included either because they constitute minor parameterizations of the evaluated backbones or because they do not introduce distinct architectural principles relevant to fine motor pose estimation. The selected set therefore captures a representative cross-section of architectural diversity while maintaining feasibility and interpretability of comparisons.

The ResNet family represents classical deep residual networks in increasing depth, enabling strong feature extraction through skip connections that facilitate the learning of complex postural configurations [15]. MobileNetV2 and EfficientNet variants prioritize computational efficiency, using depthwise-separable convolutions and compound scaling to achieve favourable accuracy–parameter trade-offs, making them suitable for resource-limited environments. In contrast, the DLCRNet models were specifically developed within the DeepLabCut framework to enhance pose estimation under challenging conditions. DLCRNet_ms5 integrates multi-scale feature extraction to improve robustness to occlusions and small-structure tracking, while the stride-16 and stride-32 variants adjust internal resolution to trade spatial precision for faster inference. Together, these nine architectures provide a comprehensive basis for assessing how network depth, multi-scale processing, parameterization, and computational footprint influence pose-estimation performance in a fine motor task requiring subpixel accuracy.

The evaluated architectures also differ substantially in terms of model capacity and effective receptive field. Approximate parameter counts range from lightweight architectures such as MobileNetV2 (≈3–4 million parameters) and EfficientNet-B0 (≈5 million parameters), to deeper residual networks such as ResNet-50 (≈25 million), ResNet-101 (≈44 million), and ResNet-152 (≈60 million parameters). DLCRNet architectures fall within an intermediate-to-high parameter range, reflecting the additional complexity introduced by multi-scale feature aggregation.

Beyond parameter count, receptive field characteristics constitute a key differentiating factor between architectures. Classical backbones such as ResNet, MobileNet, and EfficientNet rely primarily on hierarchical single-scale feature extraction, in which the effective receptive field increases progressively with network depth. In contrast, DLCRNet architectures explicitly integrate multi-scale representations, enabling simultaneous access to local fine-grained features and broader contextual information. This design is particularly advantageous for pose estimation tasks involving occlusions, limb–object interactions, and fine motor control, as it allows the network to disambiguate keypoint locations using both local appearance cues and global spatial context.

### 2.5. Performance Evaluation

To comprehensively assess the performance of each CNN architecture, a standardized evaluation framework incorporating six complementary metrics was implemented. Spatial accuracy was quantified using the root mean square error (RMSE) in pixels, which measures the average distance between predicted and ground truth keypoint locations and provides a fundamental assessment of model precision.

In addition, the percentage of correct keypoints within a 5-pixel threshold (PCK@5 px) was computed to evaluate each model’s ability to achieve accuracy levels relevant for fine motor tracking [14]. Mean average precision (mAP) across multiple thresholds was also calculated to provide an overall assessment of localization performance [18].

Computational performance was evaluated using empirically measured inference speed, expressed in frames per second (FPS), and GPU memory usage (VRAM), as these metrics directly reflect real-world deployment constraints in typical neuroscience laboratory settings. While theoretical measures such as FLOPs or parameter counts provide complementary insights, effective computational cost within the DeepLabCut framework is strongly influenced by implementation-specific factors, including multi-scale processing and stride configuration. Consequently, empirically measured metrics were considered more informative for practical use. FPS indicates the model’s suitability for real-time applications, whereas VRAM reflects the computational resources required for deployment.

To address the specific challenges posed by the staircase task, an occlusion robustness score ranging from 1 (poor robustness) to 5 (high robustness) was defined to assess each model’s ability to maintain accurate tracking when critical keypoints were partially obscured. Occlusion robustness scores were assigned independently by two raters on an overlapping subset of trials, with agreement within one score level observed in over 90% of cases.

All performance metrics were statistically compared across network architectures using appropriate inferential tests. Assumptions of normality were verified prior to parametric testing. Normally distributed variables were analyzed using one-way analysis of variance (ANOVA) followed by Tukey’s post hoc tests, whereas non-parametric distributions were evaluated using the Kruskal–Wallis test with Dunn’s correction for multiple comparisons. Ninety-five percent confidence intervals were computed for each metric to assess variability and reliability, and effect sizes (Cohen’s d or η^2^, as appropriate) were reported when relevant.

## 3. Results

### 3.1. Overall Performance of CNN Architectures

As shown in Table 2, the nine evaluated architectures exhibited clearly differentiated performance in tracking digit keypoints and pellets in the side-view Montoya staircase task. Overall performance trends were consistent across control and M1-lesioned mice, with no qualitative reordering of architecture rankings observed between experimental groups (see Section 4.4 for a discussion of limitations). This suggests that the relative performance of the evaluated architectures is robust to lesion-induced alterations in limb kinematics.

Overall, DLCRNet multi-scale architectures clearly outperform traditional architectures based on ResNet, MobileNet, or EfficientNet. DLCRNet_ms5 achieves the highest spatial accuracy, with an average RMSE of 2.8 pixels, a PCK@5 px of 95.9%, and an mAP score of 91.07%. DLCRNet_stride16_ms5 achieves very similar performance (RMSE = 2.93 px; PCK@5 px = 95.15%; mAP = 90.84%) while offering a more favourable computational trade-off. In contrast, MobileNetV2 has the lowest performance, particularly on digital keypoints, with an RMSE of 3.58 px and a PCK@5 px of 90.7%, which limits its use for tasks requiring high spatial precision. A representative qualitative comparison of keypoint trajectories in M1-lesioned mice tracked using DLCRNet_ms5 and ResNet-50 is provided in Appendix A Figure A1.

### 3.2. Spatial Accuracy: RMSE, PCK@5 px, and mAP

**Figure 2:**
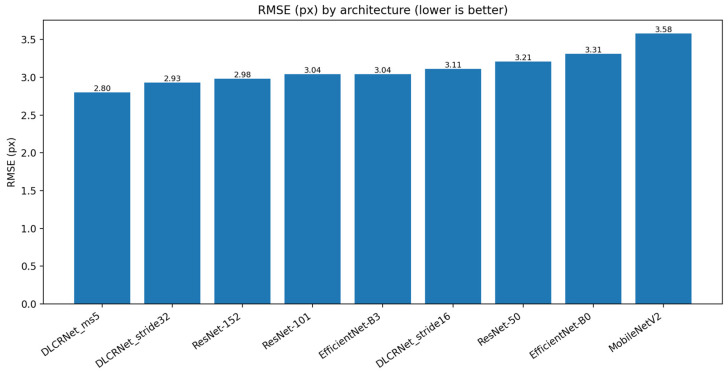
Spatial accuracy across CNN architectures measured by root mean square error (RMSE).

Performance in terms of RMSE (Figure 2) demonstrates the clear superiority of DLCRNet architectures. While ResNet-50 to 152 vary between 2.93 and 3.21 pixels of average error, the DLCRNet-ms5 and stride16 networks fall below 3 pixels, which is crucial for tracking small objects such as fingers or pellets. EfficientNet models show intermediate accuracy, with EfficientNet-B3 achieving an RMSE of 3.04 px and a PCK@5 px of 94.8%, reflecting increased sensitivity to micro-movements but slight fragility in the face of contrast variations [13].

**Figure 3:**
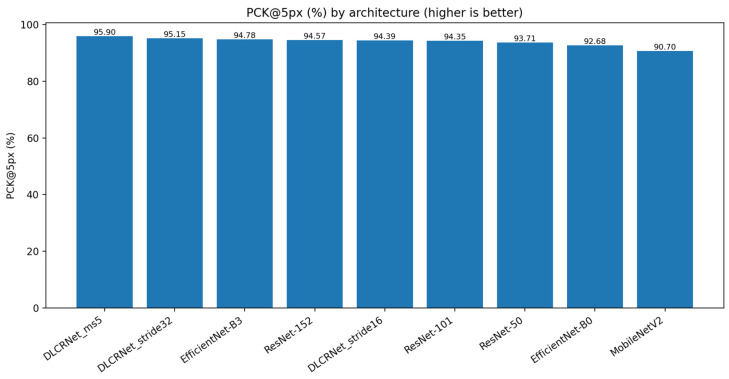
Percentage of correct keypoints within a 5-pixel threshold (PCK@5 px) for each CNN.

PCK@5 px (Figure 3) highlights the advantage of DLCRNet models in critical areas of fine movement. DLCRNet_ms5 achieves a PCK of 95.9%, outperforming all ResNet and EfficientNet models. ResNet-50, used as a baseline, offers solid performance (PCK = 93.7%), but is insufficient to capture digital details subject to occlusion in the side view. MobileNetV2 achieves the lowest score (90.7%), confirming its inability to provide accuracy compatible with kinematic analysis of fine grasping.

**Figure 4:**
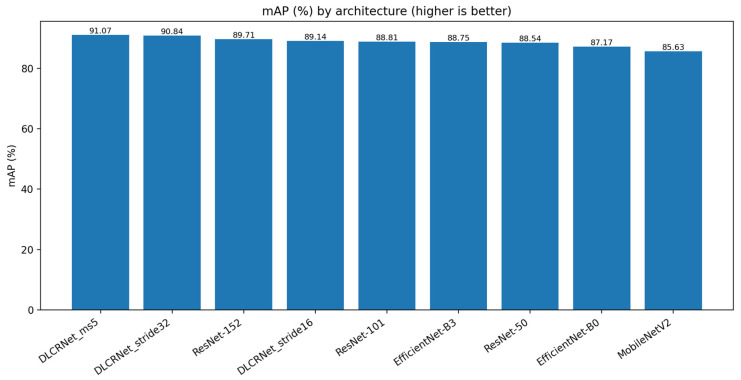
Mean average precision (mAP) across localisation thresholds for the evaluated CNN architectures.

Consistently, the mAP score (Figure 4) confirms the superiority of the DLCRNet_ms5 model (91.07%), followed closely by stride16 (90.84%). ResNet architectures plateau between 88.5% and 89.7%, while MobileNet and EfficientNet-B0 remain below 86%, reflecting less robust performance on strict accuracy thresholds.

### 3.3. Robustness to Occlusions

**Figure 5:**
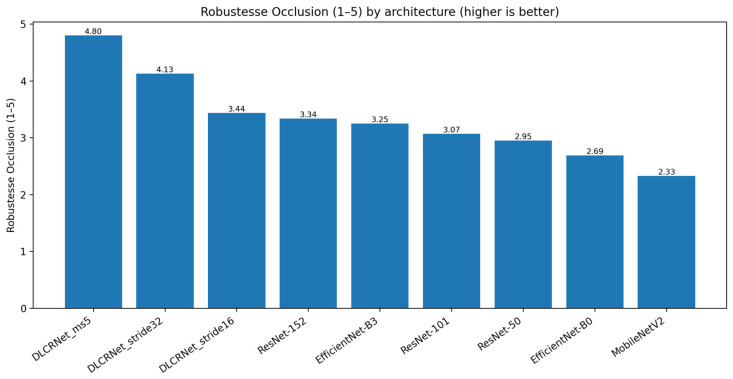
Robustness to occlusions during the Montoya staircase task across CNN architectures.

Robustness to occlusions (Figure 5) is an essential criterion in the staircase task, as fingers and the pellet frequently disappear behind the steps. The results show that DLCRNet multi-scale models are by far the most resistant: DLCRNet_ms5 scores 4.8/5, followed by DLCRNet_stride16_ms5 (4.13/5). In contrast, MobileNetV2 (2.33/5) and EfficientNet-B0 (2.69/5) frequently lose digital keypoints during lateral occlusion, compromising pellet contact detection.

ResNet models show intermediate robustness (between 2.9 and 3.34/5), which is sufficient for overall movements but insufficient to capture digital opening or pellet slippage when visibility is reduced. EfficientNet-B3 achieves adequate robustness (3.25/5), but lags behind DLCRNet models, which, thanks to their native multi-scale processing, maintain spatial consistency even in cases of partial masking.

### 3.4. Computational Cost: FPS and VRAM

**Figure 6:**
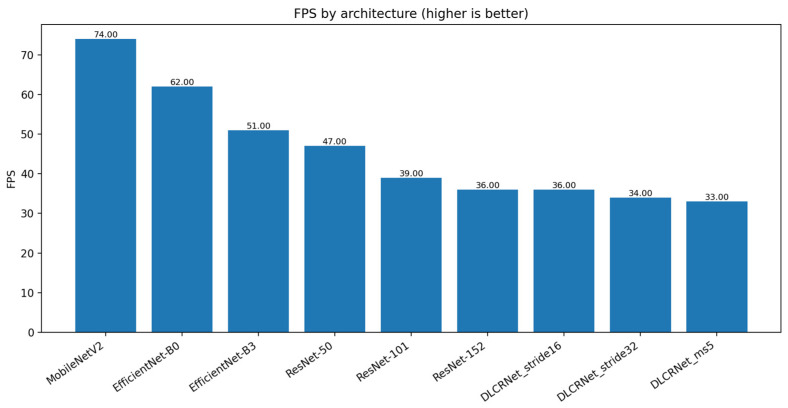
Inference speed (frames per second, FPS) measured for each CNN architecture.

**Figure 7:**
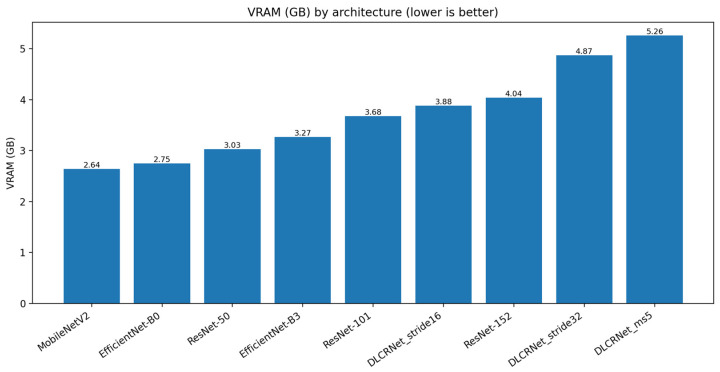
GPU memory usage (VRAM) required by each CNN architecture during inference.

Analysis of the computational cost (Figure 6 and Figure 7) highlights significant trade-offs between speed, memory, and accuracy. Lightweight architectures such as MobileNetV2 achieve high speeds (74 FPS) and a low memory footprint (2.64 GB VRAM), but at the cost of insufficient accuracy for fine motion analysis. Conversely, deep architectures such as ResNet-152 or DLCRNet_ms5 require more memory (up to 5.26 GB) and offer modest FPS (36 FPS), but produce the best spatial and kinematic estimates.

DLCRNet_stride16_ms5 stands out as an interesting compromise, combining accuracy almost equivalent to the ms5 model with slightly higher throughput (34 FPS) and more moderate memory usage (4.87 GB). This model appears to be particularly well suited to analyses requiring volume processing without sacrificing the finesse of digital tracking.

### 3.5. Comparative Summary

The combination of the three key metrics (RMSE, PCK@5 px, Occlusion Robustness) reveals a clear ranking of architectures for the staircase task. DLCRNet_ms5 is the best-performing model, but also the most expensive. DLCRNet_stride16_ms5 represents the best overall compromise, providing excellent accuracy while remaining usable on standard computing machines. EfficientNet-B3 offers an interesting alternative if the image is of high quality and has little noise. ResNet models, although stable and predictable, fail to reliably capture fingers during occlusions. Finally, MobileNetV2 does not meet the accuracy requirements for kinematic analysis of reaching.

**Figure 8:**
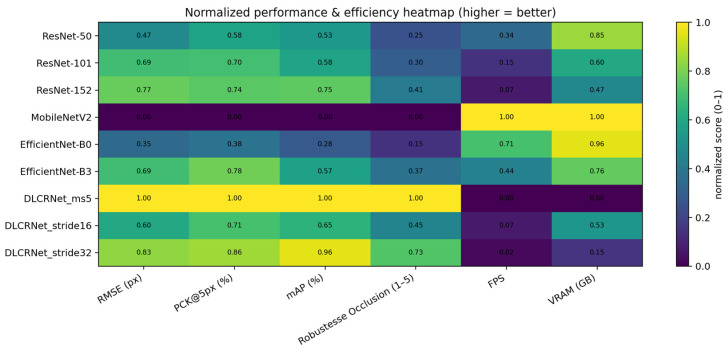
Normalized summary heatmap of all performance metrics across CNN architectures. Metrics include RMSE, PCK@5 px, occlusion robustness, inference speed (FPS), and GPU memory usage (VRAM), normalized between 0 (worst) and 1 (best).

These observations, illustrated on Figure 8, confirm that multi-scale architectures are best suited to capture micro-movements of the fingers in a task where visibility is intermittent.

## 4. Discussion

In this study, we performed a systematic comparison of convolutional neural network architectures within the DeepLabCut framework to identify optimal trade-offs between accuracy, robustness, and computational efficiency for quantifying fine motor behaviour in the mouse Montoya staircase task. Our results clearly demonstrate that CNN architecture selection has a decisive impact on pose estimation performance in complex behavioural assays, particularly those involving small distal joints and frequent occlusions. Among the evaluated models, multi-scale DLCRNet architectures, specifically DLCRNet_ms5 and DLCRNet_stride16_ms5, consistently outperformed classical backbones across most performance metrics.

### 4.1. Architecture Performance and Trade-Offs

The superior performance of DLCRNet architectures highlights the importance of multi-scale feature extraction for tasks requiring sub-pixel localization accuracy. DLCRNet_ms5 achieved the lowest RMSE and the highest PCK@5 px and mAP values, demonstrating its ability to resolve fine-grained kinematic features such as digit opening, grasp execution, and pellet manipulation. These results confirm previous observations that multi-scale representations improve robustness to occlusions and small-structure tracking, which are common challenges in skilled reaching tasks [17].

However, this increase in accuracy comes at the cost of higher computational demands. DLCRNet_ms5 required substantially more GPU memory and exhibited lower inference speed compared to lightweight architectures. In contrast, DLCRNet_stride16_ms5 provided a more favourable balance between precision and efficiency, achieving near-optimal accuracy while maintaining computational requirements compatible with standard laboratory workstations. This trade-off is particularly relevant for large-scale video analyses or longitudinal studies where processing speed and hardware constraints are critical considerations.

Classical ResNet architectures exhibited stable and predictable performance, but their accuracy plateaued despite increasing depth. Although deeper variants such as ResNet-152 marginally improved spatial precision, they failed to match the robustness of multi-scale architectures under occlusion-heavy conditions. Lightweight models, including MobileNetV2 and EfficientNet-B0, achieved high inference speeds and low memory footprints, but their reduced sensitivity to fine digit movements and frequent keypoint loss during occlusions limit their suitability for high-precision kinematic analyses. These findings underscore the intrinsic trade-offs between efficiency and accuracy previously reported for compact CNN architectures [12,13].

### 4.2. Implications for Neurobehavioural Research

The Montoya staircase task represents a stringent benchmark for pose estimation due to its requirement for millimetric precision and its high incidence of self-occlusion. Our results demonstrate that only multi-scale DLCRNet models reliably maintain stable digit trajectories under these conditions. This capability is particularly important for preclinical neuroscience studies, where subtle motor impairments often serve as sensitive indicators of neural circuit dysfunction or recovery following injury.

In models of stroke, traumatic brain injury, or neurodegenerative disease, impairments in digit coordination, reach amplitude, and grasp stability are key behavioural hallmarks [3,4]. Our analysis shows that DLCRNet_ms5 and DLCRNet_stride16_ms5 were uniquely capable of detecting kinematic signatures associated with such deficits, including reduced digit extension and increased pellet slippage. This level of sensitivity is essential for translating behavioural measurements into meaningful insights about neural function and therapeutic efficacy.

More broadly, these findings reinforce the importance of selecting pose estimation architectures that are matched to the complexity of the behavioural task under investigation. While lightweight models may be sufficient for gross locomotor analyses or low-occlusion scenarios, fine motor assays demand architectures that prioritize spatial precision and robustness over raw computational speed.

### 4.3. Broader Impact and Future Directions

Beyond the Montoya staircase task, the present results provide generalizable guidance for selecting CNN architectures in a wide range of behavioural paradigms requiring high-resolution kinematic analysis. Multi-scale architectures may offer similar advantages in other contexts involving complex limb coordination, social interactions, or multi-animal tracking scenarios [18]. Furthermore, the computational efficiency of DLCRNet_stride16_ms5 suggests potential applications in near–real-time behavioural monitoring, where rapid feedback could inform experimental or clinical decision-making.

Future work could explore several avenues to further enhance pose estimation performance. Integrating additional sensory modalities, such as depth imaging or inertial measurement units, may help disambiguate occluded keypoints and improve robustness in challenging visual conditions. Extending these approaches to other species [15] or to human kinematic analysis [19] could also broaden their translational relevance. Finally, the development of automated or semi-automated annotation pipelines may reduce the manual effort required for training high-performance models, facilitating wider adoption in neuroscience laboratories.

Beyond architectural comparisons within the DeepLabCut framework, it is important to situate DLCRNet relative to other markerless pose estimation tools reported in the literature. Several alternative frameworks, such as LEAP [20], SLEAP [21], and adaptations of OpenPose or HRNet-based approaches [22], have demonstrated strong performance in animal pose estimation tasks, particularly in settings involving simpler behaviours or reduced occlusion. However, many of these approaches rely on single-scale feature extraction or require task-specific tuning to maintain robustness under complex movement patterns.

DLCRNet was specifically developed within the DeepLabCut ecosystem to address challenges commonly encountered in behavioural neuroscience, including frequent occlusions, fine-scale limb interactions, and variability in animal posture. Its multi-scale design enables improved integration of contextual and local features, which is particularly advantageous for fine motor tasks such as the Montoya staircase test. While direct experimental comparisons with alternative frameworks were beyond the scope of the present study, the consistently strong performance of DLCRNet observed here aligns with previous reports highlighting the benefits of multi-scale architectures for robust pose estimation in challenging behavioural paradigms.

### 4.4. Limitations and Considerations

While this study provides a systematic comparison of CNN architectures for markerless pose estimation in a complex fine motor task, several limitations should be acknowledged.

First, although both control and M1-lesioned mice were included, the primary objective of this work was to evaluate relative architectural performance rather than to perform an exhaustive kinematic characterization of lesion-induced motor deficits. Consequently, subgroup-specific statistical analyses focusing on detailed biomechanical parameters were beyond the scope of the present study. Importantly, overall performance trends and architecture rankings remained consistent across control and M1-lesioned animals, indicating that the comparative conclusions are robust to lesion-induced alterations in limb kinematics.

Second, computational performance was evaluated using a single GPU configuration to ensure standardized and fair comparisons across architectures. While absolute inference speed and memory usage may vary with different hardware, batch sizes, or image resolutions, relative differences between architectures are expected to generalize across typical laboratory setups.

Third, occlusion robustness was assessed using a task-specific qualitative score designed to capture frequent partial occlusions inherent to the staircase paradigm. Although this metric does not replace a full quantitative occlusion analysis, it provides a practical and interpretable measure of model robustness in a challenging behavioural context.

**Figure A2:**
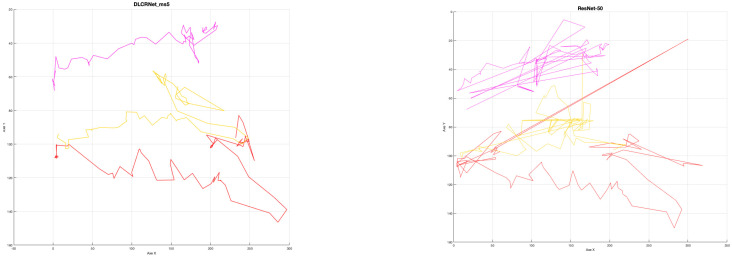
Representative keypoint trajectories in M1-lesioned mice across CNN architectures. Trajectories of the upper arm (pink), elbow (yellow) and wrist (red) during a staircase reaching trial tracked using DLCRNet_ms5 and ResNet-50. DLCRNet_ms5, showing smoother and more stable trajectories with DLCRNet, illustrating qualitative differences in robustness under lesion-induced kinematic alterations.

Finally, the supplementary qualitative comparison of keypoint trajectories in M1-lesioned mice (Appendix A Figure A2) illustrates how architecture choice can influence tracking stability under pathological conditions. These observations are intended to complement the quantitative results rather than to introduce additional statistical claims.

Together, these limitations define the intended scope of the study: a practical, architecture-focused benchmark aimed at guiding model selection for high-precision behavioural tracking rather than a comprehensive analysis of motor impairment.

## 5. Conclusions

In this study, we conducted a systematic comparison of nine convolutional neural network architectures implemented within the DeepLabCut framework for high-precision forelimb tracking in the mouse Montoya staircase task. Our results demonstrate that CNN architecture selection has a substantial impact on pose estimation accuracy, robustness to occlusions, and computational efficiency in complex fine motor behavioural assays.

Among the evaluated models, multi-scale DLCRNet architectures, particularly DLCRNet_ms5 and DLCRNet_stride16_ms, consistently provided the highest spatial accuracy and the greatest robustness under occlusion-rich conditions. While DLCRNet_ms5 achieved the best overall performance, DLCRNet_stride16_ms5 emerged as the most practical compromise, offering near-optimal accuracy with reduced computational demands. These findings highlight the importance of multi-scale representations for reliably capturing digit-level kinematics in tasks requiring millimetric precision.

The ability of DLCRNet models to detect subtle kinematic signatures associated with motor impairments, such as reduced digit extension and impaired grasp stability, underscores their value for preclinical neuroscience research. Accurate quantification of fine motor behaviour is critical for studying neural circuit function, tracking disease progression, and evaluating the efficacy of therapeutic interventions in models of stroke, traumatic brain injury, and neurodegenerative disease.

Overall, this work provides practical methodological guidance for selecting pose estimation architectures tailored to the demands of complex behavioural paradigms. By clarifying the trade-offs between accuracy and computational efficiency, our findings contribute to the standardization of markerless pose estimation pipelines and support the rigorous, reproducible quantification of fine motor behaviour in preclinical research. Future studies may build on this framework by extending these comparisons to other tasks, species, and multimodal sensing approaches.

## Figures and Tables

**Table 1:** Comparison of compared architectures regarding the state of the art.

Model	Type	Key Architectural Characteristics	Approx. Parameters (M)	Approx. Model Complexity
ResNet-50	Standard deep residual network	Common reference backbone in DeepLabCut; good compromise between speed and accuracy	~25	Medium
ResNet-101	Deeper residual network	Improved feature extraction for complex postures; higher computational cost	~44	High
ResNet-152	Very deep residual network	High sensitivity to fine spatial details; increased memory and computation requirements	~60	Very high
MobileNetV2	Lightweight efficiency-oriented network	Depthwise-separable convolutions; low memory footprint; reduced accuracy for fine digit movements	~3.5	Low
EfficientNet-B0	Optimized compact network	Excellent accuracy-to-parameter ratio; suitable for limited hardware resources	~5.3	Low–Medium
EfficientNet-B3	Larger EfficientNet variant	Improved sensitivity to small joint movements; higher computational demand	~12	Medium
DLCRNet_ms5	Multi-scale DeepLabCut	Explicit multi-scale feature aggregation; robust to occlusions and limb interactions	~35–40	High
	architecture			
DLCRNet_stride16_ms5	Multi-scale DLCRNet with	Balanced spatial resolution and inference speed	~28–30	Medium–High
	medium stride			
DLCRNet_stride32_ms5	Multi-scale DLCRNet with	Faster inference at the expense of spatial precision	~30–32	Medium
	large stride			

**Table 2:** Metrics values for each architecture of CNN.

Architecture	RMSE (px)	PCK@5 px (%)	mAP (%)	Robustness Occlusion (1–5)	FPS	VRAM (GB)
ResNet-50	3.21	93.71	88.54	2.95	47	3.03
ResNet-101	3.04	94.35	88.81	3.07	39	3.68
ResNet-152	2.98	94.57	89.71	3.34	36	4.04
MobileNetV2	3.58	90.70	85.63	2.33	74	2.64
EfficientNet-B0	3.31	92.68	87.17	2.69	62	2.75
EfficientNet-B3	3.04	94.78	88.75	3.25	51	3.27
DLCRNet_ms5	2.80	95.90	91.07	4.80	33	5.26
DLCRNet_stride16	3.11	94.39	89.14	3.44	36	3.88
DLCRNet_stride32	2.93	95.15	90.84	4.13	34	4.87

## Data Availability

Data and codes are available from the corresponding author upon reasonable request. The data are not publicly available due to INSERM on-going project.

## Abbreviations

The following abbreviations are used in this manuscript:

DLC	DeepLabCut
CNN	Convolutional Neural Network
RMSE	Root Mean Square Error
PCK	Percentage of Correct Keypoints
mAP	mean Average Precision
FPS	Frames Per Second
